# Reliability and validity of the Persian version of patient assessment of constipation- quality of life (PAC-QOL) questionnaire 

**Published:** 2017

**Authors:** Marjan Mokhtare, Seyed-Mohammad-Sadegh Ghafoori, Mojtaba Soltani-Kermanshahi, Amir-Hosein Boghratian, Shahram Agah, Mehrdad Sheikhvatan

**Affiliations:** 1 *Assistant Professor, Colorectal Research Center, Rasoul Akram Hospital, Iran University of Medical Sciences, Tehran, Iran*; 2 *Medical Doctor, Colorectal Research Center, Medical Student Research Committee (MSRC), Faculty of Medicine, Iran University of Medical Sciences, Tehran, Iran*; 3 *Biostatistics Department, International Branch, Shahid Beheshti University of Medical Sciences, Tehran, Iran*; 4 *Colorectal Research Center, Rasoul Akram Hospital, Iran University of Medical Sciences, Tehran, Iran*; 5 *Professor, Colorectal Research Center, Rasoul Akram Hospital, Iran University of Medical Sciences, Tehran, Iran*; 6 *Clinical Research Development Center, Rasoul-e-Akram Hospital, Iran University of Medical Sciences, Tehran, Iran *

**Keywords:** Quality of life, Reliability, Validity, Persian, Constipation

## Abstract

**Aim:**

The present study aimed to assess the reliability and validity of Persian version of patient assessment of constipation: quality of life (PAC-QOL) questionnaire in Iranian patients.

**Background:**

Chronic constipation has significant effects on daily living, wellbeing and individuals' quality of life (QOL). Validated tools can help us to assessing QOL in affected ones and facilitating clinical management of them.

**Methods:**

The English version of Patient Assessment of Constipation: Quality of Life (PAC-QOL) was translated into the Persian language and was confirmed by back-translation. One hundred and forty patients with functional constipation, according Rome III criteria, completed the questionnaires .The questionnaires were analyzed using Cronbach's Alpha internal consistency score to determine the reliability. Twenty medical experts were then asked to evaluate the PAC-QOL and the results were used to calculate the Content Validity Ratio (CVR) and Content Validity Index (CVI).

**Results:**

Due to obtained value for Cronbach`s α (0.975) and also for the subscale of physical discomfort (0.930), psychosocial discomfort (0.975) and worries and concerns (0.915), the internal consistency is established. According to medical experts’ opinions, the value of CVR ranged from 0.5 to 0.8 and the value of CVI was 0.81.

**Conclusion:**

The Persian version of PAC-QOL questionnaire is shown to have acceptable reliability and validity to be used for psychometric evaluation in Iranian patients complaining of functional constipation.

## Introduction

 Chronic constipation is a common gastrointestinal complaint particularly in developed countries (1, 2). Constipation has undesirable effects on mental and physical aspect of the quality of life. According to the ROME III diagnostic criteria (3, 4), functional constipation presents as “determinedly difficult, infrequent, or incomplete defecation”, without any abdominal pain, causative underlying disease and drug consumption. (5, 6). Functional constipation occurs in up to 27% of the general population and affects all age groups, but seems to be more prevalent in women and old age groups (7). 

The prevalence of constipation is high in Iran. Though it is less than Western countries, latest alterations in behavioral and dietary life style, higher urban residency, industrialization and immobility can cause an enhance in the number of patients with constipation in the future.(8, 9).

The estimated prevalence of constipation and functional constipation in Iran was 1.4-37%, and 2.4-11.2%, respectively (10-13). 

The mean total direct and indirect costs during 6-months was approximately 147 PPP$(purchasing power parity dollars) for patients with functional constipation in Iran and higher educated patients (189.75 PPP$), age over 64 (373.42 PPP$), BMI less than 18.5 kg/m2 (510.84 PPP$), and widowed persons (258.50 PPP$) had the highest expenses (14,15).

There are very little information exist to concern about the natural history, quality of life and risk factors of constipation in Iran. Selecting the best treatment for patients with functional constipation depends on how it can relief individual symptoms and improve quality of life. The original English version of a Patient Assessment of Constipation: Quality of Life (PAC-QOL) (Copyright©PAC-QOL, 2005 Mapi Research Trust, All rights reserved) that developed and validated by Marquis et al (15) in 2005, is a self-reported questionnaire frequently used to measure QOL in patients. PAC-QOL questionnaire can facilitate clinical assessment of this complaint in patients. 

PAC-QOL questionnaire has been translated and validated in majority of different languages all around the world. The present study aim to assess the reliability and validity of the translated Persian version of PAC-QOL tool in Iranian patients. 

## Methods

**Description of PAC-QOl**

The original PAC-QOL paper contains 28 items grouped into 4 subscales covering: Worries and concerns (11 items), Physical discomfort (4 items), Psychosocial discomfort (8 items), and Satisfaction of treatment (5 items). 

A 5-point Likert response scale, ranging from 0 (Not at all / none of the time) to 4 (Extremely / All of the time), is used over a 2-week recall period (A higher score indicating worse QOL due to constipation).

Total score and the scores for each subscale were calculated according to the original PAC-QOL paper (16).

**Linguistic and semantic equivalence**

 The linguistic validation of the PAC-QOL into Persian aimed to obtain a conceptually equivalent version and easily understood by our patients.

 Forward Persian translation was done by three translators (two gastroenterologists and a professional translator). Back translation to English was performed by two other experienced translators. Slight discrepancy was found in Items 18 and an expression in this item was corrected. 

However, to complete the linguistic and cultural adaptation we decided to perform a clinical review and a cognitive debriefing with patients. Both were considered a means to test the instrument’s content validity, *i.e.*, to evidence its suitability to the specific purpose. So, for a clinical review, we first asked a committee composed by a forward translator, six gastroenterologists, a biostatistician and an epidemiologist to clinically improve the Persian translated version. This gave a minor changes in translated version to improve the clarity of the questions in Persian. The questionnaire was filled by 6 series of patients for pilot test with the purpose of finding any problem of clarity, misunderstandability and redundancy of each item (17, 18). 

**Study population**

Once piloted, the Persian version was then tested for reliability and validity. For this second phase we selected 140 patients with functional constipation (according to Rome III criteria) and age over 18 who referred to gastrointestinal clinic of Rasoul-e-Akram educational hospital. The patients over 18 years with a history of chronic constipation or evacuator disorder for at least 3 months alternately also with two or more of the following symptoms: fewer than three bowel movements (complete evacuation) in a week, lumpy and/or hard stools at least a quarter of the time, the sensation of incomplete evacuation at least a quarter of the time, and straining at defecation at least a quarter of the time were included. The patients with constipation dominant irritable bowel syndrome and/or secondary specific etiology (associated to underlying disease and drug) for constipation and inability to completing the QOL questionnaire were excluded. Four Gastroenterologists gave the questionnaires to the patients. All of participants filled informed consent form.

The sample size is considered an acceptable number for validation studies and for factor analysis (19, 20). Data were collected during a period of 5 months, starting in October 2014.

**Validation methods**

The psychometric features of the PAC-QOL were determined in terms of reliability, content validity, and construct validity as described below (21).

**Reliability**

Internal consistency and reproducibility were investigated to evaluate reliability of the PAC-QOL. Internal consistency examines the complementary nature of items by searching for contradictions and measurement errors. Internal consistency of overall score and only three subscales calculated with Cronbach’s alpha. A high positive value for Cronbach’s alpha (0.7) suggests that the PAC-QOL is scoring consistently (10). To evaluate the reproducibility, a two week test–retest study was performed on 30 patients by comparing PAC-QOL scores obtained at the time of each patient’s first visit with those at the second visit, when an evacuation proctography and/or colonic transit study were undertaken without any treatment recommended between two visits. Comparisons were made using intra-class correlation coefficients (ICC) and a high positive correlation coefficient ([0.7) was taken as evidence of reproducibility (3).

**Validity**

To test the content validity, we asked 20 medical experts to evaluate the PAC-QOL and the results were used to calculate the Content Validity Ratio (CVR) and Content Validity Index (CVI). Since the numbers of specialists were 20, Cut Point for CVR was considered to be 0.42.

To test the construct validity, we used the factor analysis from 140 patients with functional constipation. Kaiser-Meyer-Olkin (KMO) measure of adequate sampling and Bartlett’s test of sphericity were considered before analysis.

**Statistical analysis**

Mean ± SD used for data expression. Statistical analyses were performed using SPSS version 18.0 by Cronbach’s alpha, intraclass correlation analysis, F test, and independent T test. P-value<0.05 was considered significant.

**Ethics**

This study was approved by the ethics committee of Iran University of Medical Sciences (study ID NO. 1941 /documented at 19/Aug/2014). Informed consent form was completed by all participants. 

## Results

**The Sample**

172 consecutive patients presented at our clinic with a chief complaint of constipation. 32 patients were excluded from our study group because of various reasons (IBS-Constipation dominant, drug consumption, underlying disease such as diabetes mellitus), thus data from 140 patients were analyzed. All the patients had been suffered from constipation for more than 3 months. Table 1 shows the distributions of the demographic, anthropometric and socioeconomic characteristics of the patients with constipation. Accordingly, the mean (SD) of age was 40.7 (10.2) and regarding the education only 51.2% had academic education. Also 77.4 % of patients were married. The liquid consumption was not differed among females and males (P-value=0.503).

Table 2 presents the distribution of the domains of PAC-QOL in patients. Accordingly, 58.6% of patients had severe constipation.

**Feasibility**

The mean PAC-QOL completion time was 16.2 ± 2.280 minutes, ranging from 14 to 20 minutes. All items were filled. Table 3 shows the PAC-QOL domains value for patients. Our sample showed a group of patients with very severe levels of constipation. It might be due to referral center.

**Reliability**

Due to obtained value for Cronbach`s α (0.975) and also for the subscale of physical discomfort (0.930), psychosocial discomfort (0.975) and worries and concerns (0.915), the internal consistency is established. It maintains excellent even if we delete an item, as shown in Table 4. Moreover, all items showed high test-retest correlation coefficients and intra-class correlation coefficients (0.974).

**Validity**

According to medical expert’s opinions, the value of content validity ratio (CVR) ranged from 0.5 to 0.8 and the value of content validity index (CVI) was 0.81. Table 5 shows the sensitivity of PAC-QOL index over the different value of the socio-demographic variables. Accordingly, there was no significant difference of PAC-QOL index regarding gender, age, education and smoking status. We also evidenced significant lower PAC-QOL score and better health, for single and those with more liquid consumption.

 To test the construct validity, we also found Kaiser-Meyer-Olkin measure of sampling adequacy index value 0.930 and Bartlett`s index value 4843.584 (P-value< 0.001). Then we performed a principal component factor analysis and evidenced the desirable triple dimensional structure, corresponding to 83.536% of explained variance. These results confirm the PAC-QOL domains.

## Discussion

We showed strong internal consistency for the total Persian PAC-QOL score and across all three subscales in our study patients. In particular, Cronbach’s alpha coefficients in the overall score and subscales were higher than 0.9, demonstrating a high level of internal consistency.

**Table 1:** Demographic, anthropometric and socioeconomic characteristics of the patients

Variable	Value	N	%
Gender	Female Male	84 56	60.0 40.0
Age	Mean (SD) Min-Max	40.7(±10.2) 18-68	-
Education	Less than diploma Diploma University degree	43 20 77	30.7 14.3 55.0
Family Status	Single Married	33 107	23.6 76.4
Smoking Status	Yes No	17 123	12.1 87.9
Alcohol Status	Yes No	9 131	6.4 93.6
Drug Status	Yes No	18 122	12.9 87.1
Liquid Consumption Status	1-2 glass per day 2-4 glass per day 4-6 glass per day 6-8 glass per day	43 80 15 2	30.7 57.2 10.7 1.4
Activity Status	Daily activity Professional	120 20	85.7 14.3

**Table 2:** Distribution of the domains of the PAC-QOL

Domains	Items Number	Severity	Frequency	Percent
Physical Discomfort	4	Mild Moderate Severe	22 35 83	15.7 25.0 59.3
Psychosocial Discomfort	8	Mild Moderate Severe	30 26 84	21.4 18.6 60.0
Worries and Concerns	11	Mild Moderate Severe	31 27 82	22.1 19.3 58.6
Total	23	Mild Moderate Severe	30 28 82	21.4 20.0 58.6

The overall score showed good reproducibility, during 2 week interval between the first and second visits same as the original PAC_QOL version evaluation (16).

Chronic constipation has significant effects on different components of individuals' QOL such as; negative economic impact, reducing quality of life, Healthcare system disturbances, Nocebo/side-effects of medicalization .We need validated tools to assess QOL in order to facilitate clinical management of constipated patients. PAC-QOL questionnaire is a self-reported questionnaire frequently used to measure QOL in constipated patients.

**Table 3:** Inter scale reliability indicators

Question	α if item deleted	Test-retest correlation coefficient
Q1	0.972	0.663
Q2	0.972	0.677
Q3	0.972	0.839
Q4	0.973	0.830
Q5	0.971	0.867
Q6	0.971	0.896
Q7	0.972	0.844
Q8	0.971	0.867
Q9	0.972	0.944
Q10	0.972	1.000
Q11	0.971	1.000
Q12	0.971	1.000
Q13	0.971	0.951
Q14	0.972	0.992
Q15	0.972	0.978
Q16	0.972	0.975
Q17	0.972	0.910
Q18	0.984	0.823
Q19	0.972	0.932
Q20	0.972	0.967
Q21	0.972	0.982
Q22	0.972	0.844
Q23	0.973	1.000

**Table 4:** Relationship between PAC-QOL Index and socio-demographic variables

Variable	Value	PAC-QOL Index	t/F	sig
Gender	Female Male	2.974 2.722	1.532	0.128
Age	Less than 30 years Between 30 & 59 years 60 or more years	2.522 2.902 3.583	2.171	0.118
Education	Less than diploma Diploma University degree	2.931 2.927 2.827	0.197	0.822
Family Status	Single Married	2.446 3.005	3.016	0.003
Smoking Status	Yes No	2.850 2.877	0.110	0.913
Liquid Consumption Status	1-2 glass per day 2-4 glass per day 4-6 glass per day 6-8 glass per day	3.055 2.899 2.467 1.000	4.260	0.007

t: Student`s t, F: F test, sig: significance (P-value)

Up to now, PAC-QOL translated and formally validated in many languages in which it is now available (4). The original English PAC-QOL tool, were studied in France (n= 30), Netherlands (n = 33), Belgium (n= 20), and Canada (n= 55), with demonstrated internal consistency, reliability, and reproducibility (16).The number of patients were low in these studies in comparison with the original English study (n= 223). 

In this study we had not satisfaction subscale because our study was done only before treatment but the satisfaction subscales that reported in the original study was 0.66 and in the Japanese Version was 0.46.

In an evaluation study of the PAC-QOL psychometric features, Dubois et al. (5) validated a 1-point improvement in the PAC-QOL score as a relevant definition of significant response for treatment. Furthermore, in the Marquis et al. study (16) estimation of 0.5 – point change was recommended as the minimum important difference in overall score on the basis their effect size. According to these two reports, difference of <1 or 0.5 does not have clinically meaning.

Attention to concurrent validity, PAC-QOL is the best gold standard constipation-specific QOL questionnaire. The original English study, the PAC-QOL scores were compared with the “clinical severity” to assess cross-sectional validity (16).

Formal linguistic validation was performed in our study as a translation–back translation method or a linguistic consensus board in comparison with the recent Japanese PAC-QOL version validation. 

However, in translating to Persian, language cultural and lifestyle differences between the two societies were considered. 

First limitation of our study was three subscales analysis without treatment satisfaction item. However, all of the patients completed 23 questions in the Persian version of PAC-QOL questionnaire, as follows: 140 patients (100%) in the internal consistency evaluation; 30 (21%) in the test–retest.

And second limitation was a difference between our patients group with constipated general population, because Rasoul-e-Akram Hospital is a referral center.

The results of this study might be generalized to Iranian constipated patients according to other studies anywhere in the world.

The PAC-QOL, is the best gold standard constipation-specific QOL questionnaire available now. Moreover, the PAC-QOL has been used more and more frequent in many high-quality studies (6). The Persian version of PAC-QOL questionnaire had acceptable reliability and validity. It can be used for Iranian patients complaining of chronic constipation all over the world.

The availability of the PAC-QOL in a variety of languages would make it feasible and easy to compare international studies and to do multicenteral researches.

## Conflict of interests

The authors declare that they have no conflict of interest.
